# One Health tick bite exposure biomarkers against Ixodes ricinus salivary peptides

**DOI:** 10.21203/rs.3.rs-10397016/v1

**Published:** 2026-08-10

**Authors:** Alexis Dziedziech, Bruno Passet, Alice Raffetin, Anavaj Sakuntabhai, Richard Paul, Kristina Persson, Sarah I. Bonnet

**Affiliations:** Lund University; Institut Pasteur; Intercommunal Hospital of Villeneuve-Saint-Georges; Institut Pasteur - Functional Genetics of Infectious Diseases Unit; Institut Pasteur; Institut Pasteur

**Keywords:** Ticks, Tick exposure, Biomarker, Diagnostics, Surveillance, Ixodes ricinus

## Abstract

The changing distributions and activity patterns of tick populations underscore the urgent need for enhanced surveillance and risk assessment strategies for ticks and tick-borne diseases (TBDs) affecting humans and animals. Biomarkers of exposure to tick salivary peptides can be used to identify tick bite history and to monitor tick species distributions. Here, we derived peptides from previously identified immunogenic proteins, namely IrCalreticulin, IrLip1, and IrSPI. Bioinformatic predictive analysis of 3D structure and computed docking strength were used to identify candidate peptides based on predicted immunogenicity and arthropod homology. Using ELISA, several peptides distinguished sera from experimentally *Ixodes ricinus*-exposed and unexposed sheep for both IgM and IgG antibodies, as was true for clinical patient samples IgG positive for tick-borne *Borrelia* bacteria. These findings offer the potential for developing new effective strategies for the surveillance of ticks and TBDs, disease diagnostic guidance as well as assessing tick population control strategies.

## Introduction

Ticks are important vectors of pathogens affecting both humans and animals; approximately 10% of tick species are of medical or veterinary importance.^1^. There are ~ 700 known species of hard ticks in the family Ixodidae and about 200 species in the soft tick family Argasidae^2^. Globally, ticks found in regions of Northern, Central, Western and/or southern Europe include *Ixodes* spp., *Dermacentor* spp., *Rhipicephalus* spp., *Hyalomma* spp. And *Haemaphysalis* spp.^3^. Due to ongoing global changes, including climatic, environmental, and socio-economic factors, the distribution, density and activity periods of several tick species are currently changing, with some expansion into previously unsuitable regions^4^. For example, recently, both the Crimean-Congo Hemorrhagic Fever virus (CCHFV) and its vector *H. marginatum* have become established in the south of France^5^. Changes are also observed or to be expected regarding tick species distribution in countries of tropical or subtropical zones^6,7^.

Ticks can transmit over 100 zoonotic pathogens including some that are genera dependent, such as the Lyme Borreliosis (LB) bacteria *Borrelia burgdorferi sensu lato* (*s.l*.) transmitted by *Ixodes* spp., whereas other pathogens are associated with multiple tick genera including *Rickettsia* spp., *Anaplasma* spp. and *Ehrlichia* spp.^8^. *Ixodes* spp. are known to transmit more than 20 viruses, bacteria, protozoa and helminths including Tick-borne encephalitis (TBE) Virus, *B. burgdorferi* s.l., *Anaplasma phagocytophilum, Babesia* spp. and *Cercopithifilaria rugosicauda*^9,10^. The burden of disease in humans caused by many tick-borne pathogens (TBPs) is increasing and is likely underestimated^11^. In Europe, ticks are the primary vectors of concern because they transmit *Borrelia* spp. and TBE virus. However, the lack of standardized tick-borne pathogen surveillance across Europe makes the overall incidence difficult to evaluate, although several approaches have been proposed to improve standardization^12^. Among reported incidence rates, Sweden has one of the highest in Europe, with 464 cases per 100,000 people^13,14.^ In France, country heterogeneity has been reported between regions due to spatial and seasonal influence^15^. For example, in 2020, 667/100,000 was reported in Limousin (central area) compared to 11/100,000 in Poitou-Charentes (western area)^16^. Beyond Europe, reports of LB are high with the highest rates occurring in North America, with an estimated 476,000 cases being diagnosed and treated annually from 2010 to 2018^17^. In addition, ticks are the primary vectors of animal pathogens worldwide, transmitting a broad range of parasites, bacteria and viruses and posing an increasing threat to animal health, livelihoods and food security^18^. Thus, surveilling and controlling the spread of the ticks vectoring TBP is of high medical and veterinary importance.

Monitoring the risk of tick bites and associated TBPs currently relies on laborious field collections of ticks and subsequent morphological and molecular identification^19^. Indirect monitoring approaches can also be time intensive and include assessing tick infestations in domestic animals and livestock to correlate these data with human risk for tick exposure^20,21^. Risk related to ticks may be tracked through sero-diagnostic screening in humans and animals for TBPs; however, because multiple tick species can vector the same pathogens, this approach has limited specificity^20^. Developing a rapid and reliable test to detect tick exposure in blood samples would provide a more accurate measure of tick presence and would be of use for assessing tick control strategies, studying the epidemiology of TBDs, and supporting clinical differential diagnosis in suspected TBD, particularly because self-reported tick exposure correlates poorly with true tick exposure^22^. Such a test could be based on the detection of host antibodies directed against tick salivary antigens injected into the vertebrate host during the tick bite. However, the major challenge lies in identifying antigenic markers capable of discriminating among the different arthropods and tick genera to which the host has been exposed, which is particularly critical in areas with a high diversity of hematophagous arthropods^23^.

Tick saliva was first shown to elicit an immune response in Guinea pigs exposed to *Dermacentor variabilis* in 1939^24^ and has since been tested for development of antibody-based tools for detecting tick exposure. However, issues related to arthropod specificity, reproducibility and costs remain^22^. For example, antibodies raised against sonicated salivary glands from *Ixodes dammini* showed cross reactivity with other arthropod exposure^25^. Nevertheless, both unique and shared components of tick saliva from *Amblyomma americanum* and *Dermacentor variabilis* have been detected by western blot analysis, suggesting distinct protein profiles that could be targeted for the development of genus-specific tick exposure tests^26^. Since then, several recombinant proteins have been tested as potential biomarker targets including Calreticulin (CRT, see below), AV422, IxsS7, and Rs24p^27–31^. Each has its limitations, including that these candidates each required multiple tick exposures to be of utility^30–32^. For example, a recent study comparing *A. americanum*-derived CRT and AV422 as serological markers of tick bites in Tanzania (tick species include *A. variegatum, Rh. appendiculatus, Rh. sanguineus*, and *Hyalomma* spp.) found although CRT had some diagnostic value in distinguishing exposed from unexposed individuals, the antibody response to AV422 did not differ significantly between the two groups.^32^. On the other hand, immunogenic peptides derived from salivary proteins Vitogellin-2 and Lipocalin, have been identified as potential targets for host antibodies; however, these candidates have not been evaluated for cross-reactivity with sera from hosts exposed to other arthropod vectors, such as mosquitos^28,33^. Although short peptide epitopes could overcome limitations related to costly production of recombinant proteins, no candidate peptide has yet been validated for this purpose.

CRT was one of the first proteins identified as immunogenic in the tick saliva, lacking a canonical endoplasmic reticulum (ER) retention signal allowing it to be secreted into the saliva^34^. Once in the host, tick CRT has been shown to bind to C1q, although it does not appear to exert anti-complement activity^35^. Nevertheless, it has been shown that CRT may facilitate pathogen transmission by infected ticks, including pathogens such as *Babesia bigemina* and the TBE virus^36,37^. Antibodies against tick saliva CRT have therefore been proposed as potential biomarkers of tick-bite exposure^38^. Anti-CRT antibodies have been reported to persist for up to 18 months following tick exposure^27^. However, results regarding the ability of tick CRT to discriminate between bites from different tick species have been variable. For example, there appeared to be no cross-reactivity between *Ixodes ricinus* Calreticulin (IrCRT) when tested against CRT from *R. sanguineus* or *Dermacentor reticulatus*, whereas cross-reactivity was observed with CRT from *Ixodes scapularis* and *A.americanum*^38,39^. Thus, identifying specific epitopes capable of distinguishing between tick species remains a challenge.

Additional tick salivary proteins with antigenic potential for identifying exposure to tick bites were either poor candidate target antigens for tick exposure biomarkers or have not yet been tested for this purpose^40^. Our previous work has demonstrated that *Ixodes ricinus* Serine Protease Inhibitor (IrSPI) and *Ixodes ricinus* Lipocalin (IrLip1), known to play a role in tick feeding, induced an immunogenic response in both mice and sheep, although the recombinant form of these proteins tested for use in an anti-tick vaccine was not protective against tick bites^41,42^. IrSPI has the ability to repress the proliferation of CD4^+^ T lymphocytes and pro-inflammatory cytokine secretion^43^. IrLip1, by binding to histamine, serotonin and prostaglandin, modulates the host immune response, and, in ticks regulates hemostasis^44^. More recently, a novel role for lipocalins was described in the soft tick, *Ornithodoros turicata americanus*, which found that Otlip increased levels of pro-apoptotic markers in mice^45^. Given the demonstrated immunogenicity of the tick salivary proteins IrCRT, IrSPI and IrLip1, identifying small antigenic peptides derived from these proteins could facilitate the development of rapid and affordable tests for tick bite exposure.

This study aimed to identify tick-specific, genus-specific and/or species-specific immunogenic peptides suitable for use in a rapid diagnostic test. To this end, immunogenic epitopes were predicted *in silico* from the tick salivary proteins IrCRT, IrSPI and IrLip1, all of which are known to elicit antibody responses in vertebrate hosts. Selected peptides were then synthesized and evaluated using sera from experimentally tick-exposed sheep to assess their potential as markers of previous tick exposure. Tick specificity was further assessed using sera from experimentally mosquito-exposed mice. The most promising candidates were subsequently tested using clinical serum samples from patients known to be IgG-seropositive for *Borrelia* spp., to further evaluate their diagnostic potential.

## Results

### Peptide prediction and synthesis

To identify putative antigenic regions within known immunogenic tick salivary proteins, a bioinformatic pipeline was developed to select optimal peptide candidates for use in detection assays (Fig. 1A). AlphaFold 3D structures were predicted for IrCRT (Fig. 1B), IrSPI (Fig. 1C) and IrLip1 (Fig. 1D). Boxed regions indicate accessible regions for each protein, as predicted by the ligand-binding algorithm in D-I-TASSER. For IrCRT, the highlighted region (Fig. 1B) corresponds to amino acid positions 74, 105, 106, 107, 109, 111, 124, 128, 131, 135, 137, 145, 317, 318, and 319. For IrSPI (Fig. 1C), the boxed residues include positions 33, 34, 35, 36, 41, 42, 43, 44, 59, 60, 61, and 62. For IrLIP (Fig. 1D), the identified residues correspond to positions 52, 53, 54, and 59. Using the MHC II binding tool from IEDB, more than 10,000 candidate epitopes per protein were predicted, with lengths ranging from 12 to 18 amino acids. The top 20 peptides for IrSPI and the top 40 peptides for IrCRT and IrLip1were selected based on percentile rank and predicted surface accessibility from D-I-Tasser. Selected peptides were then evaluated for their binding affinity to HLA alleles using MDockPep2 and assigned a synthesis score using the PepSySco tool from the IEDB Analysis Resource. Representative docking interactions with HLA alleles are shown for IrCRT39, IrSPI01 and IrLIP30 in Fig. 1E, 1F and 1G, respectively. Peptide selection was additionally based on BLASTP searches against the NCBI non-redundant protein sequence database (https://blast.ncbi.nlm.nih.gov/Blast.cgi?PROGRAM=blastp&PAGE_TYPE=BlastSearch&LINK_LOC=blasthome). Multiple sequence alignments (MSAs) of representative peptides demonstrate varying degrees of conservations across tick genera (Supplementary Figs. 1 and 2). IrCRT39 is highly conserved across tick genera, including *Rhipicephalus* (94.1% identity), *Ixodes* (100% identity), *Dermacentor* (88.2% identity), *Haemaphysalis* (88.2% identity), *Hyalomma* (88.2% identity) and *Amblyomma* (88.2% identity, Supplementary Fig. 1 and Supplementary Fig. 3). IrSPI01 showed very limited conservation outside *I. ricinus*, with 90.9% identity to *I. scapularis* (Supplementary Fig. 2A and B and Supplementary Fig. 4A). Similarly, IrLIP04 was largely species-specific, sharing 91.7% identity with *I. scapularis*, with minimal similarity to other arthropod sequences (Supplementary Fig. 2C and D and Supplementary Fig. 4B). Based on these criteria, six peptides were selected and synthesized for both IrCRT and IrSPI, and eight peptides for IrLip1 (Table 1).

To further refine candidate regions, elongated peptides were designed based on predicted B-Cell epitopes using the BepiPred algorithm (IEDB Analysis Resource; Table 2). Regions with scores above the 0.5 threshold were considered potentially immunogenic (highlighted in yellow; see Fig. 1H, 1I, 1J for IrCRT, IrSPI, IrLip1, respectively). For IrCRT, the region spanning amino acids 17–59 (IrCRT1a) was predicted to be immunogenic. The central region (positions 73–310) displayed alternating patterns of predicted immunogenicity and was therefore not suitable for synthesis. Two additional peptides, IrCRT6a and IrCRT7a, were designed to cover the C-terminal regions spanning positions 313–349 and 380–413, respectively (Fig. 1H, Table 2). For IrSPI, a large portion of the sequence was predicted to be immunogenic. Two elongated peptides were designed to cover residues 14–49 I(rSPI1a) and 40–74 (IrSPI2a) (Fig. 1I, Table 1). In contrast, IrLip1 exhibited alternating regions of high and low predicted immunogenicity between position 35 and 300, and complicated peptide design due to aggregation propensity in the central region (Fig. 1J). Consequently, only three regions could be successfully synthesized: residues 91–128 (IrLIP2a), 188–215 (IrLIP4a) and 265–289 (IrLIP5a) (Fig. 1J, Table 1).

### ELISA for detecting host response to tick antigens in sheep

To evaluate whether the candidate peptides could discriminate between sera from animals exposed or unexposed to ticks, we used serum samples from a previous study aimed at developing an anti-tick vaccine based on IrSPI and IrLip1^42^. Using a linear mixed-effects model, we compared antibody responses in sheep before and after tick infestation. The model included infestation status as a fixed effect, and random intercepts for ELISA batch and plate. Significant increases in peptide-specific IgM and IgG responses were observed for a subset of HLA-docking–selected peptides (Fig. 2A). For IgM, significant effects of infestation were observed for CRT39, *t*(3) = 4.45, *p* = 0.021; LIP04, *t*(3) = 3.49, *p* = 0.040; and CRT12, *t*(3) = 3.48, *p* = 0.040, indicating increased post-infestation responses. For IgG, significant increases following infestation were detected for CRT42, *t*(3) = 6.06, *p* = 0.009; LIP04, *t*(3) = 4.99, *p* = 0.015; and SPI11, *t*(6) = 2.72, *p* = 0.034. Overall, these results indicate that infestation was associated with increased peptide-specific IgM and/or IgG responses for a subset of predicted immunogenetic epitopes (Table 3).

When analyzing BepiPred-derived extended peptide antigens, linear mixed-effects modeling revealed a significant increase in IgG responses following infestation for SPI1a (*t*(4) = 6.83, *p* = 0.002), indicating a clear infestation-associated antibody response (Fig. 4B, Supplementary table 1).

To further enhance peptide specificity and immunoreactivity, modifications including peptide multimerization (e.g., LIP04×3), biotinylation, and multiple antigen peptide (MAP) synthesis were tested. Although these modifications were expected to increase antibody binding by enhancing epitope density and accessibility, only the three times repeat of the LIP04 motif yielded a difference between the pre- and post-exposure sheep samples. Although it was not significant, it was similar in effect size to LIP04 alone (Supplementary Fig. 5, Supplementary table 2).

### Verifying vector specificity of tick saliva antigens

To assess whether the candidate tick antigens showed cross-reactivity with antibodies generated against mosquito salivary components, we compared antibody responses to sera exposed to known mosquitoes vectors from various genera. Mouse sera exposed to either *Aedes aegypti/Aedes albopictus/Aedes vexans/Aedes malayensis/Culex pipiens molestus* or *Anopheles stephensi* were tested against peptides CRT25, CRT39, LIP04, LIP04×3, LIP5a, SPI01 and SPI1a at a serum concentration of 1/600. Immune reactivity of sheep samples post-tick infestation was compared to all other groups (pre-infestation sheep, un-infested mice, *Aedes*/*Culex* infested mice and *Anopheles* infested mice) using a linear mixed-effects model. We found that infested sheep were much more significantly likely to react against the tick salivary peptides in both IgM and IgG conditions (Fig. 3). The peptides eliciting the strongest IgM responses to tick-infested sheep serum compared to all non-tick infested serum included: LIP04 t(27.5) = 8.17, p < 0.001, SPI1a t(23.5) = 7.71, p < 0.001, LIP04×3 t(17.0) = 5.98, p < 0.001, LIP5a t(17.1) = 5.87, p < 0.001, and SPI01 t(19.6) = 4.94, p < 0.001. CRT39 also reached significance t(18.0) = 2.73, p = 0.024 (Table 4). The strongest IgG responses were observed against: LIP04 t(28.1) = 5.47, p < 0.001, followed by SPI1a t(23.5) = 3.88, p = 0.001, LIP5a t(21.7) = 2.59, p = 0.026, and LIP04×3 t(21.4) = 2.39, p = 0.036 (Table 4). Of note, SPI01 showed a borderline trend. Overall, these results support the vector specificity for ticks over mosquitos for the candidate peptides LIP04, LIP04×3, LIP5a and SPI1a against both IgM and IgG antibodies.

### Response to tick peptides in *Borrelia*-infected patients

To evaluate whether the candidate peptides could discriminate between sera from patients clinically diagnosed with *Borrelia* infection, we tested serum samples that were either IgG-positive or IgG-negative for *Borrelia*. Using a linear mixed-effects model, we compared IgM and IgG antibody responses between the positive and negative groups. The model included infection status as a fixed effect and random intercepts for ELISA batch and plate. Compared with the IgG-negative group, patients who were IgG-positive for *Borrelia* showed significantly stronger IgG responses to several peptides, whereas no significant differences were observed for IgM responses (Fig. 4A). Specifically, significantly higher IgG reactivity was detected against the peptides CRT39 (t(42.8) = 3.24, p = 0.034, CRT42 (t(42.9) = 2.75,p = 0.034, SPI11 (t(43.7) = 2.98, p = 0.034), SPI1a (t(43.0) = 2.83, p = 0.034), and LIP04 (t(43.9) = 2.52, p = 0.0497, (Table 5). Notably, the peptides LIP5a, SPI01, and LIP04×3 showed trends toward significance, with p-values slightly above the 0.05 threshold.

Next, the *Borrelia* IgG-positive patients were stratified into three groups according to their anti-*Borrelia* IgG titers 0 < IgG ≤ 0.8 (low), 0.8 < IgG ≤ 3 (medium), IgG > 3 (high). No significant differences in IgM responses to candidate tick salivary peptides were observed when comparing uninfected patients with the low-, medium-, or high-titer groups. However, patients with the lowest anti-*Borrelia* IgG titers exhibited the strongest IgM responses to the candidate peptides, with the most pronounced difference observed for LIP04 (Fig. 2B, left). In contrast, analysis of IgG responses to the candidate tick salivary peptides revealed significant differences for selected peptides in both the medium- and high-titer groups (Fig. 2B). Compared with uninfected patients, the medium-titer group showed significantly higher IgG reactivity to CRT42 *t*(42) = 3.36, *p* = 0.010 and CRT39 *t*(42) = 2.89, *p* = 0.036 (Table 6). The high-titer group exhibited significantly higher IgG reactivity to SPI11 *t*(41,6) = 3.43, *p* = 0.008, LIP04 *t*(41,6) = 3.36, *p* = 0.010, LIP04×3 *t*(40.20) = 2.91, *p* = 0.036, and CRT39 *t*(40,1) = 2.54, *p* = 0.036 (Table 6). In addition, SPI11 in the medium-titer group and SPI1a in both the medium- and high-titer groups showed trends toward significance, with p-values slightly above the 0.05 threshold. Taken together, these results indicate that patients with low anti-*Borrelia* IgG titers exhibited the strongest IgM responses and the weakest IgG responses to the candidate tick salivary peptides. SPI11, LIP04 and CRT39 were the most effective peptides for discriminating infected from uninfected patients in the medium- and high-titer groups, followed by LIP04×3 and CRT42, while SPI01 and SPI1a demonstrated discriminatory potential.

## Discussion

In this study, we synthesized peptides derived from known immunogenic *I. ricinus* salivary proteins, IrCRT, IrSPI and IrLip1 based on IEDB predictions, with the aim of developing short, reliable peptide antigens for the detection of tick exposure. ELISA screening identified several immunoreactive epitopes, of which four peptides emerged as the most promising candidates based on their ability to discriminate between tick-exposed and unexposed sera for both IgM and IgG detection. Among the short peptides (8–15 amino acids), IrLIP04 and IrSPI11 showed the best overall discriminatory performance. Among the extended peptides (25–45 amino acids), IrLIP5a and IrSPI1a consistently produced the strongest performance against IgG in sheep sera. Repeating the IrLIP04 sequence three times (LIP04×3) also improve performance across serum samples and resulted in discrimination between tick-exposed and unexposed animals, although larger sample sizes will be required to confirm its statistical significance. In contrast, other peptide modifications including multiple antigen peptides (MAPs) and biotinylation did not improve antibody recognition.

When the most promising peptide antigens were evaluated using human sera from patients who were IgG-positive for *Borrelia*, results were consistent with those obtained in sheep. CRT39, LIP04, LIP04×3, SPI11 and SPI1a were the best recognized peptides by antibodies following a tick bite. Moreover, stratification of patients according to their anti-*Borrelia* IgG titers revealed that individuals with medium and high titers also displayed the strongest IgG responses against the tick salivary peptides. One possible explanation is that these patients had been infected for a longer period, allowing sufficient time for the development of an adaptive immune response to tick saliva. Previous studies have shown that anti-tick salivary IgG responses generally require approximately 3–7 weeks to develop following tick exposure^46^. Consistent with this hypothesis, although not statistically significant, patients with the lowest anti-*Borrelia* IgG titers exhibited the strongest IgM responses but the weakest IgG responses against the candidate tick peptides. This pattern is compatible with the expected transition from an early IgM-dominated response to a more mature IgG response following tick exposure. Together, these findings suggest that antibody responses to tick salivary antigens evolve dynamically during *Borrelia* infection and support the potential utility of combining IgM and IgG measurements when assessing recent versus past tick exposure. It should be noted that the present study was limited by the number of available sera, reflecting the ethical constraints associated with experimental tick infestations. Overall, the peptide antigens developed here are short, scalable, and compatible with antibody-based diagnostic platforms, including multiplex assays, rapid diagnostic tests, and low-cost ELISA formats, in contrast to the full recombinant immunogenic proteins such as IrCRT, IrSPI and IrLip1^27,42^.

The candidate antigens were validated using sera from sheep and humans exposed to *I. ricinus* supporting their potential application within a One Health framework. Multiple sequence alignments suggest that peptides derived from IrSPI and IrLIP may provide greater specificity for *Ixodes* spp. exposure, whereas CRT-derived peptides could potentially detect exposure across several tick genera. However, species-level specificity remains to be established through testing against sera from hosts exposed to additional tick species. For example, calreticulin has been reported to be a poor marker of exposure to *Haemaphysalis* species, whereas immunogenic calreticulin has been described in *A. americanum, Dermacentor variabilis, I. scapularis* and *I. ricinus*^22,47^. Furthermore, tick salivary composition undergoes extensive “sialome switching” throughout blood feeding and is influenced by tick developmental stage and infection status, resulting in marked differences in the salivary proteins expressed under different biological conditions^40^. Taking advantage of the peculiarities of each sialome composition could aid in the future development of species-specific or genus-specific peptide antigen identification. Future studies should therefore investigate whether the peptides identified here, together with newly identified candidates, can discriminate not only between tick-exposed and unexposed hosts but also between exposure to different tick genera or species.

Because animal hosts and infectious agents do not respect geographic or ecological boundaries, robust tick exposure monitoring tools could have significant public health value by supporting a standardized surveillance of emerging tick-associated risks. For example, in Europe, tracking the spread of *Hyalomma marginatum* is of major One Health relevance, particularly given the recent detection of Crimean-Congo hemorrhagic fever virus (CCHFV) in established tick populations in southern France^5^. As ticks expand their geographic range and pathogens emerge in new regions under environmental and climatic pressures, it is essential that public health agencies strengthen surveillance and adapt prevention strategies through monitoring of tick populations, pathogen circulation, and risk awareness campaigns.

More broadly, the epidemiology of zoonotic pathogens transmitted by arthropod vectors is a complex issue that requires an understanding of the multipartite system, namely the vector(s), the pathogen(s) and the reservoir host(s). Research and surveillance efforts have historically focused primarily on human infections, despite humans representing only one component of this transmission network. A One Health approach, integrating animal hosts into surveillance frameworks, is therefore essential for improving our understanding of pathogen transmission dynamics^3^. A standardized serological assay for measuring tick exposure, such as the one proposed in the present work, could facilitate monitoring of tick population expansion and associated pathogens, improve assessment of health risks in both humans and animals, identify transmission hotspots and evaluate the effectiveness of tick control programs including acaricide-based interventions. Such tools may ultimately contribute to more targeted vector control strategies while reducing unnecessary acaricide use, thereby limiting impacts on biodiversity, environmental health, and the development of acaricide resistance.

## Materials and Methods

### Serum collection from hosts exposed to arthropod vectors

Sheep blood samples have been collected from four seven-month-old male sheep (PreAlps breed) as previously described^42^. Briefly, blood samples were collected in 10 mL vacutainer tubes from the jugular vein at day 0 and day 28 following infestation with laboratory-reared *I. ricinus*. Approximatively 1,000 larvae and 48 nymphs (42% infected with *Anaplasma phagocytophilum*) were allowed to feed on each sheep until repletion. Sera were obtained following centrifugation and stored at − 20°C until ELISA analysis.

Serum samples from mosquito-exposed SWISS mice, were kindly provided by Anna-Bella Failloux and the CEPIA platform at the Institut Pasteur, (Paris, France). For unexposed mice, blood samples were collected from three one-month-old naive mice. For mosquito-exposed mice (three months old), three animals were repeatedly exposed over a two-month period to *Aedes aegypti, Aedes albopictus*, *Aedes vexans*, *Aedes malayensis*, and *Culex pipiens molestus*. After centrifugation, sera were collected and stored at − 20°C until use.

Human sera that tested either positive or negative for *Borrelia burgdorferi* sensu lato, the causative agent of LB, were obtained from, Reference Center for Tick-Borne Disease at the Intercommunal Hospital of Villeneuve-Saint-Georges, France. Thirty serum samples from patients who were IgG-positive for *Borrelia* and had a history of tick exposure were compared with 14 serum samples from patients who were IgG-negative for *Borrelia* and had no known history of tick exposure. All serum samples were anonymized and originally collected as part of routine clinical care. An information sheet describing the secondary use of clinical samples for research was systematically provided to all patients attending the hospital. Only samples from patients who had provided consent for research use were included in this study. The study sponsor declared compliance with the “Reference Methodology MR004” of the French Data Protection Authority (CNIL, 2,216,096 v 0, December 10, 2019). Sera samples were diluted 1:100 in the appropriate assay buffer and stored at − 80°C until analysis.

### Bioinformatic identification and production of peptides

Both IrSPI (GenBank accession number: W6HZ31.2) and IrLip1 (GenBank accession number: QOJ01963.1) have been identified and evaluated as immunogenic salivary components, as previously described^41–43^. IrCRT (GenBank accession number: AAR29958.1), has been identified as a secreted salivary protein from *Amblyomma* and *Dermacentor* ticks and has been shown to play an important role during tick feeding, with highly variable intronic sequences across tick species^34,48^.

Peptide selection based on the sequence of these three proteins was performed following the bioinformatic workflow presented in Fig. 1A. Predicted three-dimensional (3D) structures of the selected peptides were generated using the AlphaFold3 Server^49^. After removal of signal peptide sequences using SignalIP-5.0, short peptides (8–17 amino acids in length) were selected based on their predicted immunogenicity using the Immune Epitope Database (IEDB)^50,51^. Initial selection of candidate peptides was performed using the MHC class II binding prediction tool from IEDB which identifies epitopes with a high likelihood of presentation to CD4 + T helper cells using the NETMHCIIpan 4.1 EL(recommended peptide predictor) against all 27 Human HLA alleles available in the Immune Epitope Database (IEDB) Analysis Resource^50,51^. These MHC class II short peptide candidates were derived from *I. ricinus* salivary proteins IrCRT, IrSPI and IrLIP. Peptide–HLA binding affinity was further evaluated using MDockPep2^52^. Predicted 3D structures of HLA alleles were obtained from the pHLA3D database^28,53^, and docking interactions were visualized using Unipro UGENE^54^. Peptides were subsequently assessed for their protein-protein interaction accessibility based on their tertiary structure using the D-I-TASSER online server^55,56^. Each peptide was additionally analyzed using NCBI Blast to confirm sequence specificity to *Ixodes* or other tick genera including *Amblyomma, Dermacentor, Haemaphysalis, Hyalomma*, and *Rhipicephalus*^57^. Selected peptides were finally evaluated based on their “Synthesis Score” calculated using IEDB^50,51^ before final synthesis by GenScript (Piscataway, New Jersey, USA).

Following the initial ELISA screening of the synthesized short peptides, extended peptide sequences or modified versions of the most promising candidates were designed to enhance antibody binding to sera from tick-exposed hosts (Fig. 1A). Extended peptides were designed based on predictions from the Bepipred linear epitope 2.0 tool from IEDB, which identifies B-cell epitopes, resulting in peptide lengths of approximately 25–45 amino acids^50,51^. Modified peptides included the addition of biotin tags and/or the generation of multiple antigenic peptides (MAPs), in which four copies of the same peptide are assembled on a lysine-based backbone (approximately 20 amino acids per branch, see Table 2). All peptides were subsequently re-evaluated by ELISA using sera from hosts exposed to either ticks or mosquitos (Fig. 1A).

### Enzyme Linked Immunosorbent Assay (ELISA)

Polystyrene 96-well ELISA plates (MaxiSorb, NUNC, Roskilde, Danemark) were coated with 100 μL per well of either 0.4 μg/mL of peptides or 10 μg/mL of recombinant proteins diluted in carbonate–bicarbonate buffer (pH 9.6, Sigma-Aldrich, Darmstadt, Germany). For biotinylated peptides, Streptavidin-coated plates (EvenCoat, Biotechne CP004) were used. Plates were incubated at 37°C for two hours and then washed three times with 250 μL of PBS containing 0.05% Tween 20 (Montanox 20, Seppic, France). Plates were subsequently blocked overnight at room temperature with PBS containing 2% Bovine Serum Albumin (Sigma). After washing, serum samples diluted in PBS (1/100 and 1/600), were added in duplicate and incubated for 2 hours at room temperature. Following washing, plates were incubated for 1 hour at room temperature with the appropriate horseradish peroxidase (HRP)-conjugated secondary antibodies: rabbit anti-sheep IgM (1:3,000; Bio-Techne), rabbit anti-sheep IgG (1:3,000; Thermo Fisher Scientific), goat anti-mouse IgM (1:6,000; Thermo Fisher Scientific), or goat anti-mouse IgG1 (1:6,000; Thermo Fisher Scientific) depending on the host species. The reaction was revealed by adding 1- Step Slow 3,3′,5,5′-tetramethylbenzidine (TMB, Fisher Scientific) for 20 min and stopped with 50 μL of 2N H_2_SO_4_. Optical density was measured at 450 nm using an ELISA plate reader (Tecan).

### Statistical analysis, Phylogenies and Data Visualization

Statistical analyses were performed using R (v4.1). Differences in ELISA responses between pre- and post-infestation samples in sheep and uninfected and *Borrelia*-infected samples in humans were assessed using linear mixed-effects models fitted with the lme4 package^58^. Infestation status was included as a fixed effect, while ELISA batch and plate number were treated as random effects to account for nonspecific experimental variation. Data were log 10 -transformed using log(x + 1) to reduce skewness. For unpaired samples in the mosquito-exposed mice and rabbit datasets, batch preparation was included as an additional random effect. Data processing, annotation, and visualization were performed using Tidyverse, dyplr and ggplot2 packages. To improve model fit, a log(x + 1) transformation was once again applied prior to analysis. P-values were adjusted for multiple comparisons using the Benjamini–Hochberg false discovery rate.^59^ Pairwise sequence alignments were performed using the PAM30 matrix in the NCBI blast tool, optimized for short sequences. Alignment results corresponding to each peptide motif were visualized in UGene. Phylogenetic trees were constructed in UGene using the PHYLIP Neighbor-Joining method. All graphics and figures were made using Adobe Illustrator CS6.

## Supplementary Material

This is a list of supplementary files associated with this preprint. Click to download.

- Supplementaryfigurefile.docx

## Figures and Tables

**Figure 1:**
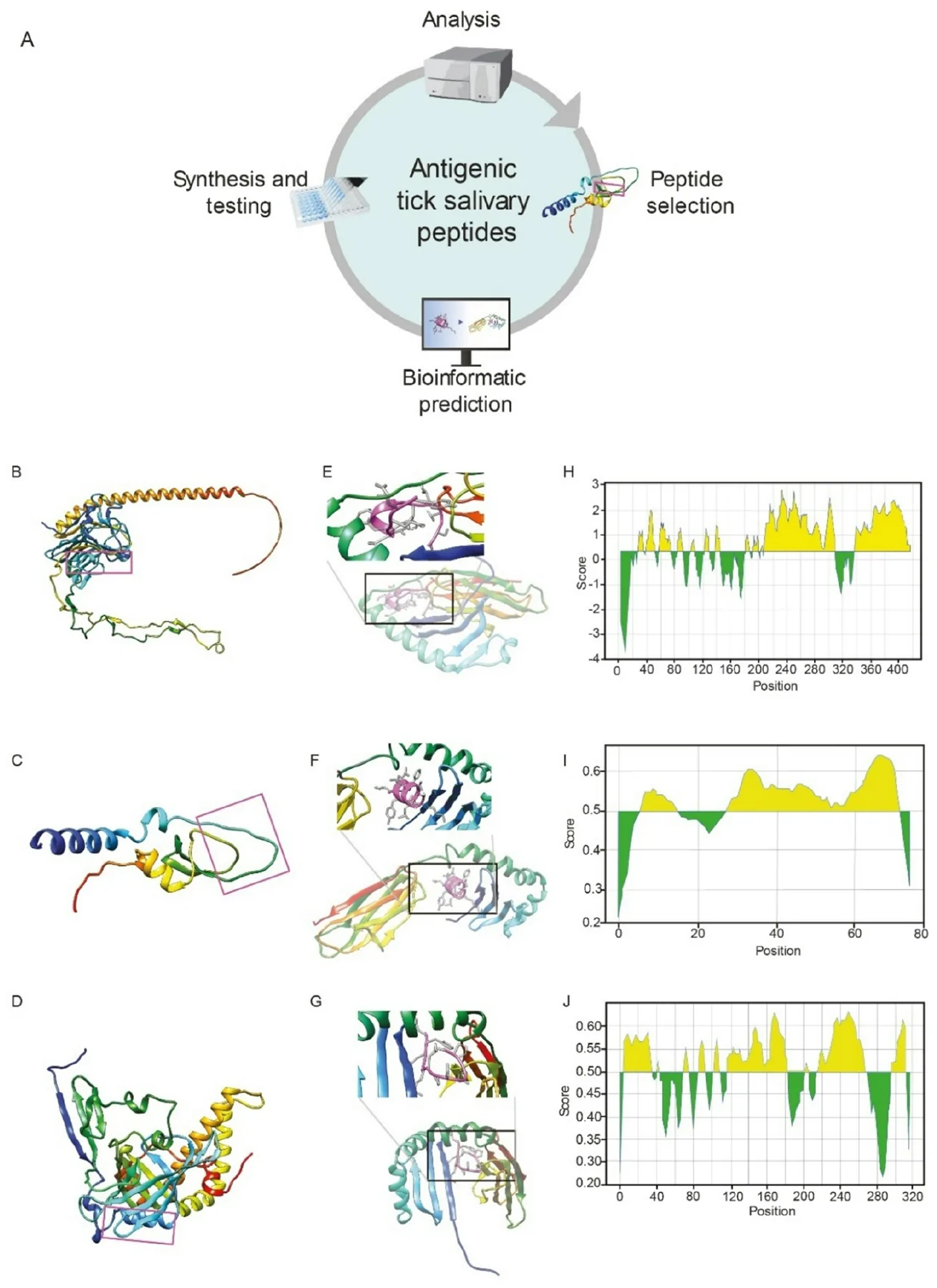
Bioinformatic pipeline for predicting immunogenic regions *I. ricinus* antigenic peptides. (a) Schematic representing the bioinformatic workflow for selecting peptides *in silico*, testing synthesized peptides with ELISA, and then analyzing the results to determine how best to modify or proceed with each peptide. (b) 3D structure of IrCalreticulin with the pink box showing the I-TASSER prediction for binding. (c) 3D structure of IrSPI with the pink box showing the I-TASSER prediction for binding. (d) 3D structure of IrLipocalin with the pink box showing the I-TASSER prediction for binding. (e) Docked structures of T cell epitope CRT39 against the MHCII receptor HLA allele HLA-DQA1*05:01/DQB1*02:01, black box is zoomed in region. (f) Docked structures of T cell epitope SPI01 against the MHCII receptor HLA allele HLA-DRB1*15:01, black box is zoomed in region. (g) Docked structures of T cell epitope LIP04 against the MHCII receptor HLA allele HLA-DRB1*01:01, black box is zoomed in region. (h) B cell antigenicity scores along the linearized salivary protein sequences for IrCalreticulin using BepiPred 2.0 from IEDB. (i) B cell antigenicity scores along the linearized salivary protein sequences for IrSPI using BepiPred 2.0 from IEDB. (j) B cell antigenicity scores along the linearized salivary protein sequences for IrLipocalin using BepiPred 2.0 from IEDB. Proteins are colored from the N-terminus (blue) until the C-terminus (red). Positive antigenicity scores are colored yellow while non-antigenic regions are colored green.

**Figure 2:**
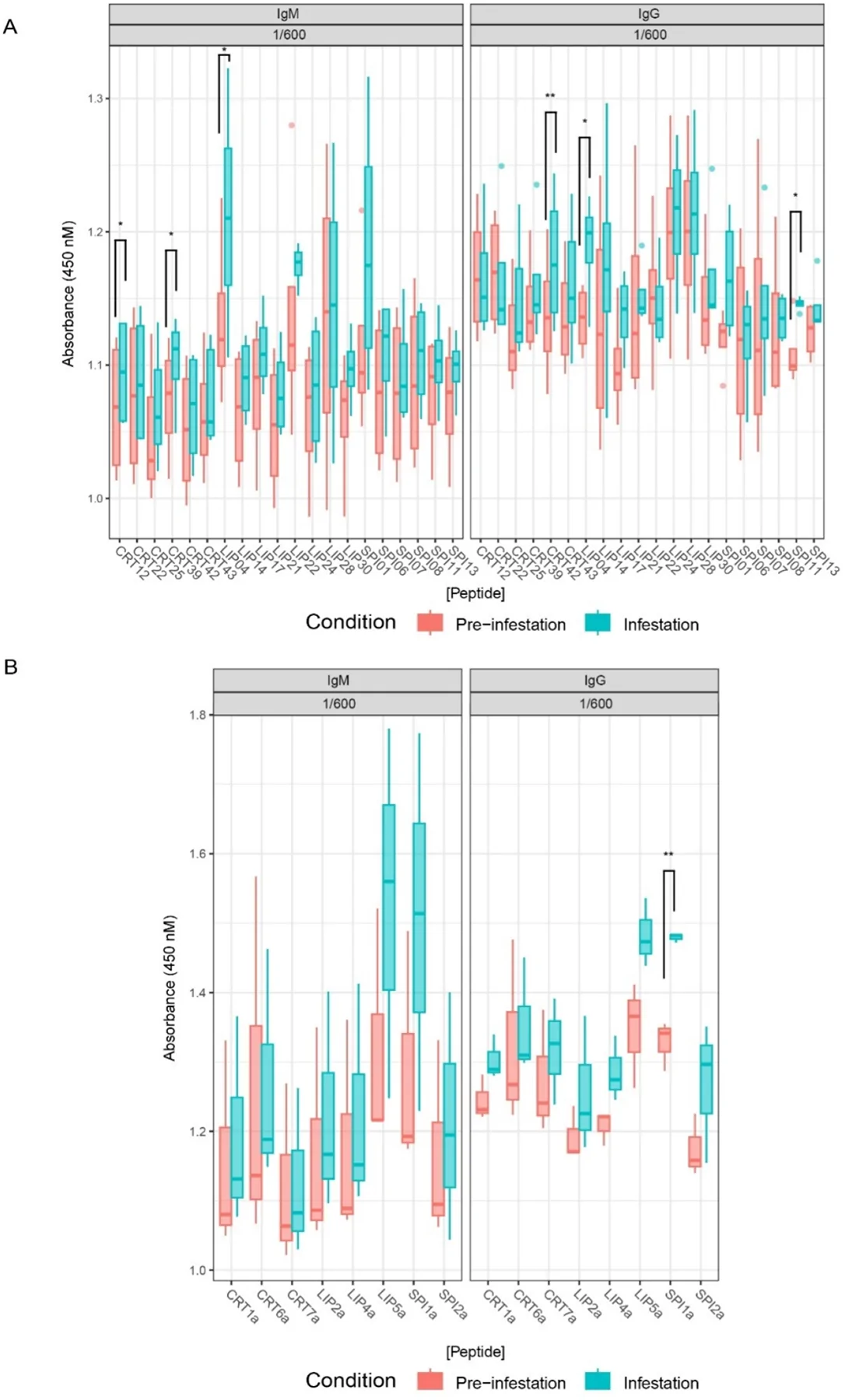
IgM and IgG antibody response of exposed tick sera to candidate tick saliva peptides Pre- (Red) and post-infestation (blue) tick sera from sheep analyzed with ELISAs for determining the antigenicity of predicted immune epitopes. (a) Boxplots show ELISA absorbance values (450 nm) for individual peptides based on HLA epitopes. Responses were measured at a 1:600 serum dilution from sheep exposed to the *Ixodes ricinus* tick, separated by antibody isotype (IgM, left; IgG, right). Peptides are listed along the X axis and were analyzed using linear mixed-effects models (LMMs) with infestation status as a fixed effect and random intercepts to account for repeated measures and technical variation (sample ID, ELISA batch, and plate). Boxes indicate the interquartile range with the median; whiskers extend to 1.5×IQR. (b) Boxplots show ELISA absorbance values (450 nm) for individual peptides based on B-Cell epitopes. Responses were measured at a 1:600 serum dilution, separated by antibody isotype (IgM, left; IgG, right). Peptides are listed along the X axis and were analyzed using linear mixed-effects models (LMMs) with infestation status as a fixed effect and random intercepts to account for repeated measures and technical variation (sample ID, ELISA batch, and plate). Boxes indicate the interquartile range with the median; whiskers extend to 1.5×IQR. Asterisks indicate peptides with a significant difference between pre- and post-infestation responses (* = *p* < 0.05, ** = *p* < 0.01, *** = *p* < 0.001).

**Figure 3:**
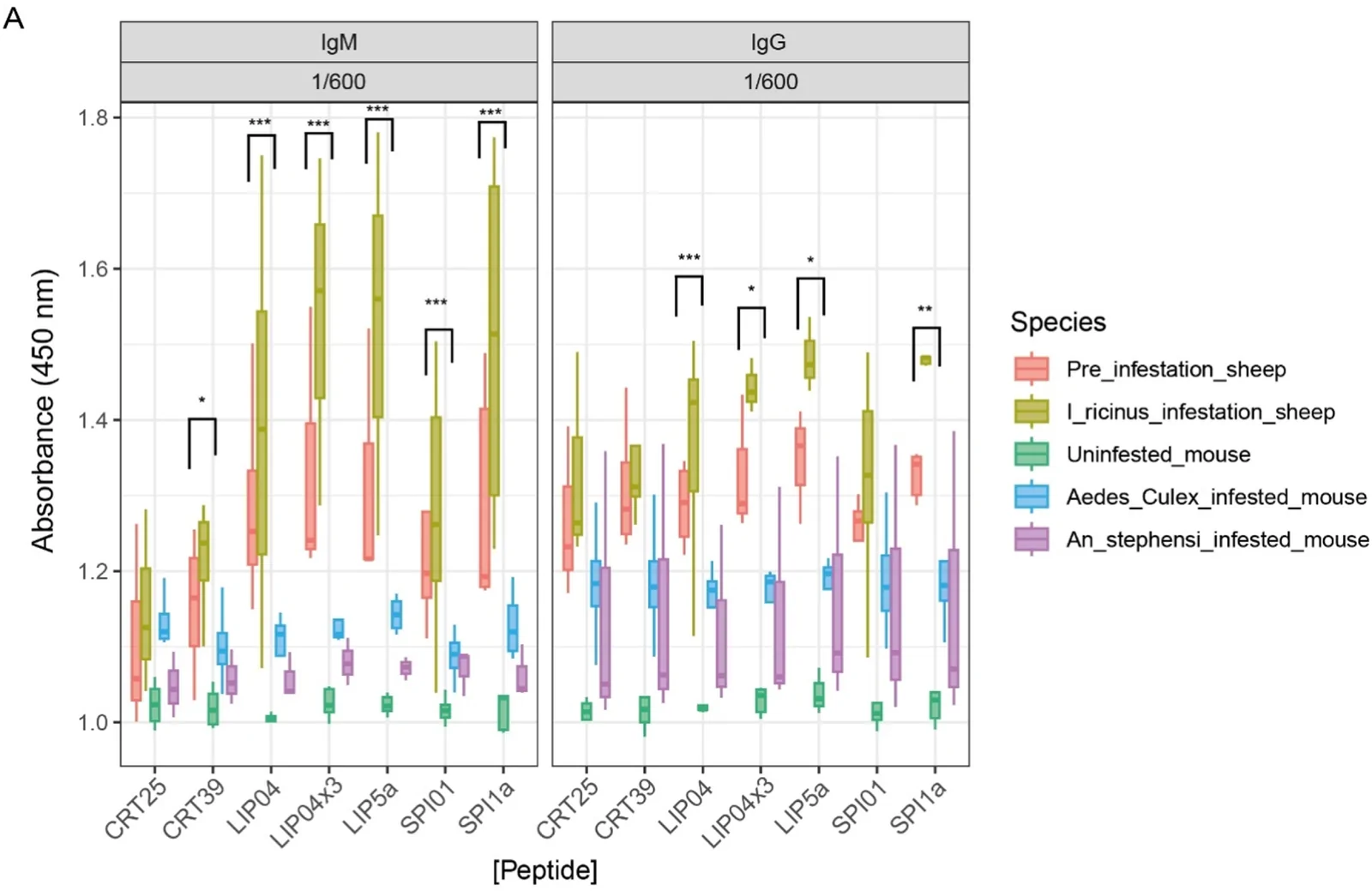
IgM and IgG antibody cross-reactivity response of sera exposed to either mosquito or ticks to candidate tick saliva peptides Comparison of *I. ricinus*infested sheep sera (yellow-green) against either pre-tick exposed sheep sera (red), non-exposed mice sera (green), *Aedes/Culex*-exposed mice sera (blue) or *Anopheles stephensi* mosquito-exposed mice sera (purple). Boxplots show ELISA absorbance values (450 nm) for individual peptides. Responses were measured at a 1:600 serum dilution and separated by antibody isotype (IgM, left; IgG, right). Peptides are listed along the X axis and were analyzed using linear mixed-effects models (LMMs) with infestation status as a fixed effect and random intercepts to account for technical variation (ELISA batch, and plate). Boxes indicate the interquartile range with the median; whiskers extend to 1.5×IQR. Asterisks indicate peptides with a significant difference between pre- and post-infestation responses (* = *p* < 0.05, ** = *p* < 0.01, *** = *p* < 0.001).

**Figure 4:**
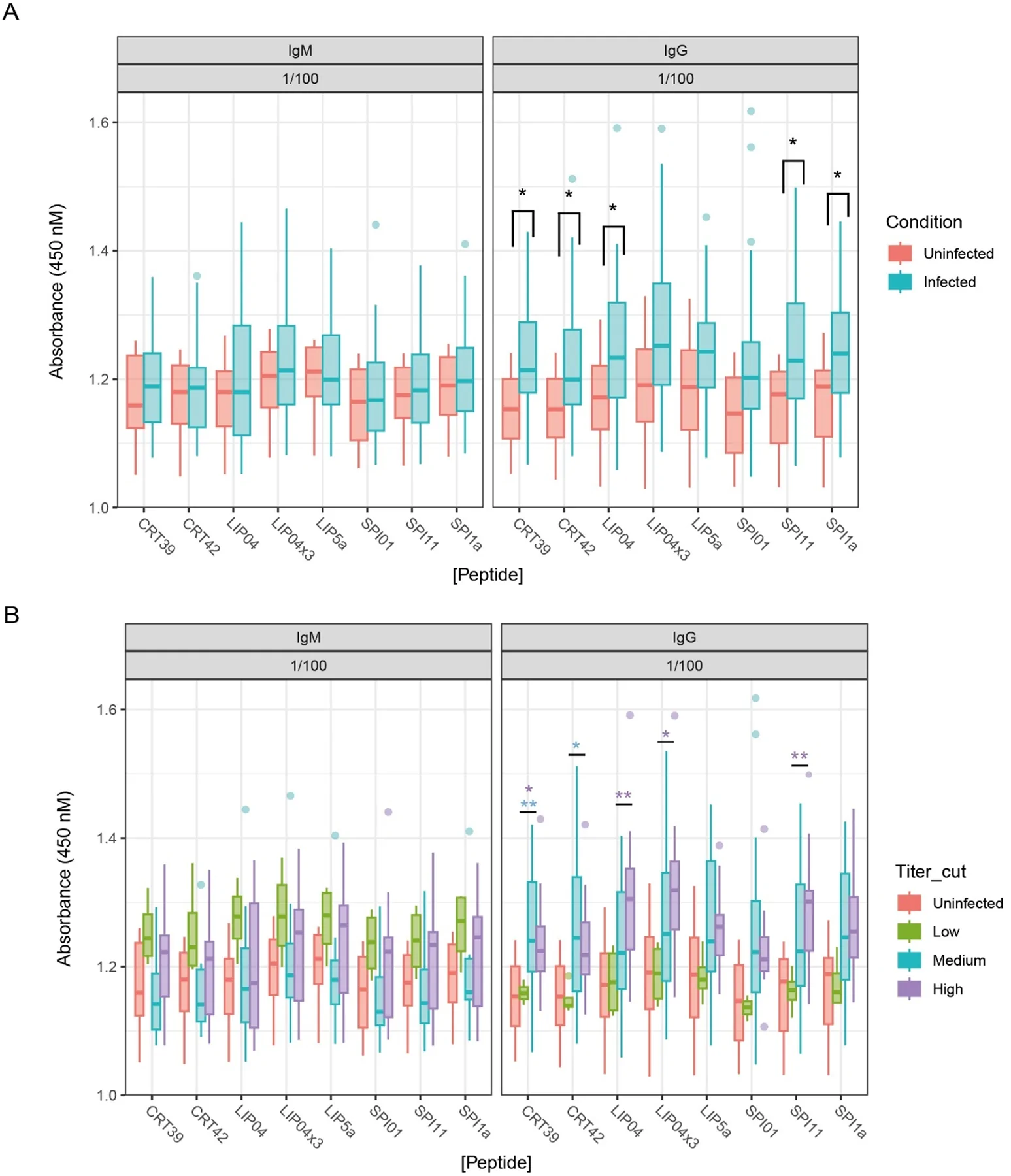
IgM and IgG antibody responses in human patients IgG positive for Borrelia tested against *I. ricinus* tick salivary antigenic peptides (a) Uninfected (Red) and IgG positive for Borrelia Infected (blue) human patients analyzed with ELISAs for determining the antigenicity of predicted immune epitopes. Boxplots show ELISA absorbance values (450 nm) for individual peptides based on selected tick saliva peptides. Responses were measured at a 1:100 serum dilution from humans with presumed exposure to the *I. ricinus* tick based on having a positive IgG result for Borrelia, and separated by antibody isotype (IgM, left; IgG, right). Peptides are listed along the X axis and were analyzed using linear mixed-effects models (LMMs) with Borrelia infection status as a fixed effect and random intercepts to account for repeated measures and technical variation (ELISA batch, and plate). Boxes indicate the interquartile range with the median; whiskers extend to 1.5×IQR. (b) Uninfected (Red) and Borrelia infected patients analyzed with ELISAs for determining the antigenicity of predicted immune epitopes. Positively infected samples were divided into three groups, either low > 0 (green), medium > 0.8 (blue) or high > 3 (purple) based on their IgG Borrelia titers. Boxplots show ELISA absorbance values (450 nm) for individual peptides based on selected tick saliva peptides. Responses were measured at a 1:100 serum dilution and are separated by antibody isotype (IgM, left; IgG, right). Peptides are listed along the X axis and were analyzed using linear mixed-effects models (LMMs) with Borrelia infection status as a fixed effect and random intercepts to account for repeated measures and technical variation (ELISA batch, and plate). Boxes indicate the interquartile range with the median; whiskers extend to 1.5×IQR. Asterisks indicate peptides with a significant difference between low, medium (blue asterisks), and high (purple asterisks) titer groups (* = *p* < 0.05, ** = *p* < 0.01, *** = *p* < 0.001).

**Table 1:** Selected candidate peptides based on the MHCII receptor function in the Immune Epitope Database (IEDB). Peptides listed come from the *I. ricinus* protein sequences, Calreticulin (CRT), Serine Protease Inhibitor (SPI) and Lipocalin (LIP), proteins secreted during tick feeding

Protein	Name	Peptide sequence	length	IEDB method	Predicted HLA Allele interaction	PepSySco
Calreticulin	CRT42	GTFKLSAGKFHGD	13	MHC-II Binding Predictions	HLA-DRB5*01:01	0,92316
Calreticulin	CRT39	KSGTIFDNILVTDDEEY	17	MHC-II Binding Predictions	HLA-DQA1*05:01/ DQB1*02:01	0,85123
Calreticulin	CRT12	FDNILVTDDEEYA	13	MHC-II Binding Predictions	HLA-DQA1*05:01/ DQB1*02:01	0,93977
Calreticulin	CRT25	EQGTFKLSAGKFH	13	MHC-II Binding Predictions	HLA-DRB5*01:01	0,95647
Calreticulin	CRT22	DEEAPAAGDAEEE	13	MHC-II Binding Predictions	HLA-DQA1*03:01/ DQB1*03:02	0,95484
Calreticulin	CRT43	GRVFADPKVYLKE	13	MHC-II Binding Predictions	HLA-DRB1*03:01	0,96517
Lipocalin	LIP30	YENTTIQVRAL	11	MHC-II Binding Predictions	HLA-DQA1*01:02/ DQB1*06:02	0,99695
Lipocalin	LIP21	IERWFQDVVWK	11	MHC-II Binding Predictions	HLA-DQA1*01:01/ DQB1*05:01	0,97178
Lipocalin	LIP17	CYILRSPPALG	11	MHC-II Binding Predictions	HLA-DRB1*01:01	0,96312
Lipocalin	LIP04	NFKGMLPNATPV	12	MHC-II Binding Predictions	HLA-DRB1*01:01	0,9777
Lipocalin	LIP14	QCYILRSPPALG	12	MHC-II Binding Predictions	HLA-DRB1*01:01	0,88208
Lipocalin	LIP28	DDIERWFQDVVWK	13	MHC-II Binding Predictions	HLA-DQA1*01:01/ DQB1*05:01	0,9307
Lipocalin	LIP22	RANFKGMLPNATPVP	15	MHC-II Binding Predictions	HLA-DRB1*01:01	0,8392
Lipocalin	LIP24	KRISLDCVLAFMI	13	MHC-II Binding Predictions	HLA-DRB3*01:01	0,7105
SPI	SPI11	RTVYSFNNNTN	11	MHC-II Binding Predictions	HLA-DRB1*04:05	0,99722
SPI	SPI13	SCAEGAKLRTV	11	MHC-II Binding Predictions	HLA-DQA1*05:01/ DQB1*03:01	0,99601
SPI	SPI06	RTVYSFNNNTNQ	12	MHC-II Binding Predictions	HLA-DRB1*04:01	0,98836
SPI	SPI01	AKLRTVYSFNN	11	MHC-II Binding Predictions	HLA-DRB1*15:01	0,99695
SPI	SPI8	RTVYSFNNNTNQCEPL	16	MHC-II Binding Predictions	HLA-DRB3*02:02	0,90001
SPI	SPI7	TVYSFNNNTNQC	12	MHC-II Binding Predictions	HLA-DRB1*04:01	0,98447

**Table 2:** Selected candidate peptides based on the B-Cell prediction of epitopes using the Bepipred function in the Immune Epitope Database (IEDB). Peptides listed come from the *I. ricinus* protein sequences, Calreticulin (CRT), Serine Protease Inhibitor (SPI) and Lipocalin (LIP), proteins secreted during tick feeding.

Protein	Name	Peptide sequence	length	IEDB method	Notes
SPI	SPI1a	SCAEGAKLRTVYSFNNNTNQCEPLKG	36	Antibody Epitope Prediction (Bepipred)	Elongated
SPI	SPI2a	SCGEGVNQFETEDCCRRECPYGKHSKVGKVSDGTF	35	Antibody Epitope Prediction (Bepipred)	Elongated
Lipocalin	LIP2a	YENTTIQVRALNQTDYPLENVIRANFKGMLPNATPVPR	38	Antibody Epitope Prediction (Bepipred)	Elongated
Lipocalin	LIP4a	YVVYNEPQCYILRSPPALGGCDLWLRKT	28	Antibody Epitope Prediction (Bepipred)	Elongated
Lipocalin	LIP5a	DDIERWFQDVVWKRISLDCVLAFMI	25	Antibody Epitope Prediction (Bepipred)	Elongated
Calreticulin	CRT1a	DPKVYLKEEFADGDAWTSRWVHSPKKGSEQGTFKLSAGKFHGD	43	Antibody Epitope Prediction (Bepipred)	Elongated
Calreticulin	CRT6a	TLGFDLWQVKSGTIFDNILVTDDEEYARQYGEETWGA	37	Antibody Epitope Prediction (Bepipred)	Elongated
Calreticulin	CRT7a	EDFEEEDEEDKKEDEEAPAAGDAEEEHKHDHEEL	34	Antibody Epitope Prediction (Bepipred)	Elongated

**Table 3:** Sheep-*I. ricinus* exposed tick sera IgM and IgG response to short *I. ricinus* salivary antigenic peptides derived from predicted MHCII interactions and assessed by linear mixed modelling.

Immuno-globulin	Peptide	effect	Comparison	estimate	std.error	statistic	df	adj p.value	signif
IgM	CRT39	fixed	Infested vs Pre-infested	0,05	0,01	4,45	3,00	0,02	*
IgM	LIP04	fixed	Infested vs Pre-infested	0,13	0,04	3,49	3,00	0,04	*
IgM	CRT12	fixed	Infested vs Pre-infested	0,05	0,01	3,48	3,00	0,04	*
IgM	SPI01	fixed	Infested vs Pre-infested	0,12	0,05	2,59	3,00	0,08	.
IgM	LIP30	fixed	Infested vs Pre-infested	0,07	0,03	2,47	3,00	0,09	.
IgM	LIP21	fixed	Infested vs Pre-infested	0,05	0,02	2,41	3,00	0,10	.
IgM	CRT43	fixed	Infested vs Pre-infested	0,03	0,01	2,11	4,15	0,10	.
IgM	LIP17	fixed	Infested vs Pre-infested	0,06	0,03	2,10	3,00	0,13	ns
IgM	LIP14	fixed	Infested vs Pre-infested	0,05	0,02	2,07	3,00	0,13	ns
IgM	LIP24	fixed	Infested vs Pre-infested	0,04	0,02	1,87	3,00	0,16	ns
IgM	CRT42	fixed	Infested vs Pre-infested	0,03	0,02	1,60	4,03	0,18	ns
IgM	SPI11	fixed	Infested vs Pre-infested	0,05	0,03	1,65	3,00	0,20	ns
IgM	SPI06	fixed	Infested vs Pre-infested	0,05	0,03	1,50	3,00	0,23	ns
IgM	SPI13	fixed	Infested vs Pre-infested	0,04	0,03	1,43	3,00	0,25	ns
IgM	LIP28	fixed	Infested vs Pre-infested	0,02	0,02	1,31	3,00	0,28	ns
IgM	CRT25	fixed	Infested vs Pre-infested	0,04	0,03	1,18	3,00	0,32	ns
IgM	CRT22	fixed	Infested vs Pre-infested	0,02	0,02	1,17	3,00	0,33	ns
IgM	SPI08	fixed	Infested vs Pre-infested	0,03	0,03	1,01	3,00	0,39	ns
IgM	LIP22	fixed	Infested vs Pre-infested	0,07	0,07	0,94	3,72	0,40	ns
IgM	SPI07	fixed	Infested vs Pre-infested	0,03	0,04	0,94	3,00	0,42	ns
IgG	CRT42	fixed	Infested vs Pre-infested	0,07	0,01	6,06	3,00	0,01	**
IgG	LIP04	fixed	Infested vs Pre-infested	0,09	0,02	4,99	3,00	0,02	*
IgG	SPI11	fixed	Infested vs Pre-infested	0,07	0,02	2,72	6,00	0,03	*
IgG	SPI01	fixed	Infested vs Pre-infested	0,08	0,03	3,15	3,00	0,05	.
IgG	CRT25	fixed	Infested vs Pre-infested	0,05	0,01	3,86	2,00	0,06	.
IgG	LIP30	fixed	Infested vs Pre-infested	0,04	0,02	2,53	3,00	0,09	.
IgG	CRT43	fixed	Infested vs Pre-infested	0,04	0,02	1,99	5,07	0,10	ns
IgG	LIP17	fixed	Infested vs Pre-infested	0,07	0,03	2,27	3,00	0,11	ns
IgG	LIP28	fixed	Infested vs Pre-infested	0,03	0,01	2,23	3,00	0,11	ns
IgG	CRT39	fixed	Infested vs Pre-infested	0,03	0,02	1,97	3,00	0,14	ns
IgG	SPI13	fixed	Infested vs Pre-infested	0,03	0,02	1,78	3,00	0,17	ns
IgG	LIP14	fixed	Infested vs Pre-infested	0,08	0,06	1,22	4,07	0,29	ns
IgG	LIP24	fixed	Infested vs Pre-infested	0,02	0,03	0,72	4,03	0,51	ns
IgG	SPI07	fixed	Infested vs Pre-infested	0,03	0,04	0,65	3,00	0,56	ns
IgG	SPI08	fixed	Infested vs Pre-infested	0,01	0,05	0,30	3,00	0,78	ns
IgG	LIP22	fixed	Infested vs Pre-infested	−0,01	0,05	−0,21	3,00	0,85	ns
IgG	LIP21	fixed	Infested vs Pre-infested	0,01	0,05	0,20	3,00	0,85	ns
IgG	CRT22	fixed	Infested vs Pre-infested	−0,01	0,04	−0,19	3,00	0,86	ns
IgG	CRT12	fixed	Infested vs Pre-infested	0,00	0,04	−0,11	3,00	0,92	ns
IgG	SPI06	fixed	Infested vs Pre-infested	0,00	0,05	0,10	4,05	0,93	ns

**Table 4:** Infested sheep-*I. ricinus* exposed tick sera IgM and IgG response to modified *I. ricinus* salivary antigenic peptides compared to mice exposed to mosquitos (*Aedes, Culex* or *Anopheles*) and assessed with linear mixed model.

Immuno-globulin	Peptide	effect	Comparison	estimate	std.error	statistic	df	adj p.value	signif
IgM	LIP04	fixed	I_ricinus vs non I_ricinus	0,19	0,02	8,17	27,51	0,00	***
IgM	SPI1a	fixed	I_ricinus vs non I_ricinus	0,23	0,03	7,71	23,47	0,00	***
IgM	LIP04×3	fixed	I_ricinus vs non I_ricinus	0,20	0,03	5,98	17,04	0,00	***
IgM	LIP5a	fixed	I_ricinus vs non I_ricinus	0,22	0,04	5,87	17,08	0,00	***
IgM	SPI01	fixed	I_ricinus vs non I_ricinus	0,17	0,03	4,94	19,57	0,00	***
IgM	CRT39	fixed	I_ricinus vs non I_ricinus	0,07	0,02	2,73	18,00	0,02	*
IgM	CRT25	fixed	I_ricinus vs non I_ricinus	0,04	0,03	1,63	20,66	0,14	ns
IgG	LIP04	fixed	I_ricinus vs non I_ricinus	0,13	0,02	5,47	28,08	0,00	***
IgG	SPI1a	fixed	I_ricinus vs non I_ricinus	0,17	0,04	3,88	23,49	0,00	**
IgG	LIP5a	fixed	I_ricinus vs non I_ricinus	0,16	0,06	2,59	21,67	0,03	*
IgG	LIP04×3	fixed	I_ricinus vs non I_ricinus	0,13	0,06	2,39	21,35	0,04	*
IgG	SPI01	fixed	I_ricinus vs non I_ricinus	0,11	0,05	2,11	26,98	0,06	.
IgG	CRT25	fixed	I_ricinus vs non I_ricinus	0,09	0,07	1,33	21,73	0,21	ns
IgG	CRT39	fixed	I_ricinus vs non I_ricinus	0,06	0,06	1,06	22,80	0,30	ns

**Table 5:** Comparison of Borrelia IgG positive patient sera IgM and IgG response to modified *I. ricinus* salivary antigenic peptides compared against patients Borrelia negative assessed with linear mixed model.

Immuno-globulin	Peptide	effect	Comparison	estimate	std.error	statistic	df	adj p.value	signif
IgM	LIP04	fixed	Infected Vs Uninfected	0,03	0,03	1,09	44,00	0,45	ns
IgM	LIP04×3	fixed	Infected Vs Uninfected	0,03	0,03	1,10	44,00	0,45	ns
IgM	SPI1a	fixed	Infected Vs Uninfected	0,02	0,02	0,98	44,00	0,48	ns
IgM	CRT39	fixed	Infected Vs Uninfected	0,02	0,02	0,92	44,00	0,48	ns
IgM	CRT42	fixed	Infected Vs Uninfected	0,02	0,02	0,73	44,00	0,50	ns
IgM	SPI01	fixed	Infected Vs Uninfected	0,02	0,02	0,75	44,00	0,50	ns
IgM	SPI11	fixed	Infected Vs Uninfected	0,02	0,02	0,83	44,00	0,50	ns
IgM	LIP5a	fixed	Infected Vs Uninfected	0,01	0,03	0,58	44,00	0,57	ns
IgG	CRT39	fixed	Infected Vs Uninfected	0,09	0,03	3,24	42,79	0,03	*
IgG	CRT42	fixed	Infected Vs Uninfected	0,08	0,03	2,75	42,87	0,03	*
IgG	SPI11	fixed	Infected Vs Uninfected	0,09	0,03	2,98	43,75	0,03	*
IgG	SPI1a	fixed	Infected Vs Uninfected	0,08	0,03	2,83	42,95	0,03	*
IgG	LIP04	fixed	Infected Vs Uninfected	0,08	0,03	2,52	43,91	0,05	*
IgG	LIP5a	fixed	Infected Vs Uninfected	0,06	0,03	2,34	42,81	0,05	.
IgG	SPI01	fixed	Infected Vs Uninfected	0,08	0,03	2,38	41,77	0,05	.
IgG	LIP04×3	fixed	Infected Vs Uninfected	0,08	0,03	2,26	42,96	0,06	.

**Table 6:** High, medium, and low titers of Borrelia IgG positive patient sera IgM and IgG response to modified *I. ricinus* salivary antigenic peptides compared against patients Borrelia negative assessed with linear mixed model.

Immuno-globulin	Peptide	Comparison	estimate	SE	df	t.ratio	adj p.value	signif
IgM	CRT39	Uninfected vs Low	−0,09	0,04	41,65	−2,10	0,12	ns
IgM	CRT42	Uninfected vs Low	−0,09	0,04	41,65	−2,06	0,14	ns
IgM	SPI01	Uninfected vs Low	−0,08	0,04	41,65	−1,78	0,16	ns
IgM	SPI01	Uninfected vs High	−0,05	0,03	41,63	−1,58	0,18	ns
IgM	CRT39	Uninfected vs High	−0,05	0,03	41,63	−1,57	0,19	ns
IgM	SPI11	Uninfected vs Low	−0,07	0,04	41,65	−1,69	0,20	ns
IgM	SPI11	Uninfected vs High	−0,05	0,03	41,63	−1,51	0,21	ns
IgM	SPI1a	Uninfected vs Low	−0,08	0,04	41,65	−1,85	0,22	ns
IgM	LIP04	Uninfected vs Low	−0,10	0,05	41,65	−1,99	0,24	ns
IgM	LIP5a	Uninfected vs Low	−0,07	0,04	41,65	−1,60	0,28	ns
IgM	CRT42	Uninfected vs High	−0,03	0,03	41,63	−1,11	0,33	ns
IgM	LIP04×3	Uninfected vs Low	−0,09	0,05	41,65	−1,81	0,35	ns
IgM	SPI1a	Uninfected vs High	−0,04	0,03	41,63	−1,20	0,36	ns
IgM	LIP5a	Uninfected vs High	−0,03	0,03	41,63	−1,08	0,43	ns
IgM	LIP04×3	Uninfected vs High	−0,04	0,04	41,63	−1,01	0,47	ns
IgM	LIP04	Uninfected vs High	−0,04	0,04	41,63	−0,96	0,51	ns
IgM	SPI01	Uninfected vs Medium	0,01	0,03	41,21	0,51	0,61	ns
IgM	LIP5a	Uninfected vs Medium	0,01	0,03	41,21	0,41	0,69	ns
IgM	LIP04×3	Uninfected vs Medium	−0,01	0,03	41,21	−0,38	0,71	ns
IgM	LIP04	Uninfected vs Medium	−0,01	0,03	41,21	−0,36	0,72	ns
IgM	CRT42	Uninfected vs Medium	0,01	0,03	41,21	0,33	0,74	ns
IgM	CRT39	Uninfected vs Medium	0,01	0,03	41,21	0,33	0,75	ns
IgM	SPI11	Uninfected vs Medium	0,01	0,03	41,21	0,31	0,76	ns
IgM	SPI1a	Uninfected vs Medium	0,00	0,03	41,21	−0,07	0,95	ns
IgG	SPI11	Uninfected vs High	−0,13	0,04	41,63	−3,43	0,01	**
IgG	LIP04	Uninfected vs High	−0,14	0,04	41,63	−3,36	0,01	**
IgG	CRT39	Uninfected vs Medium	−0,10	0,03	41,96	−3,36	0,01	**
IgG	LIP04×3	Uninfected vs High	−0,13	0,04	40,21	−2,91	0,04	*
IgG	CRT42	Uninfected vs Medium	−0,10	0,03	41,98	−2,89	0,04	*
IgG	CRT39	Uninfected vs High	−0,09	0,03	40,15	−2,54	0,04	*
IgG	SPI11	Uninfected vs Medium	−0,08	0,04	41,21	−2,40	0,05	.
IgG	SPI1a	Uninfected vs Medium	−0,08	0,03	41,97	−2,47	0,05	.
IgG	SPI1a	Uninfected vs High	−0,10	0,04	40,76	−2,74	0,05	.
IgG	SPI01	Uninfected vs Medium	−0,11	0,04	41,71	−2,60	0,08	.
IgG	CRT42	Uninfected vs High	−0,08	0,04	40,28	−2,20	0,10	ns
IgG	LIP5a	Uninfected vs Medium	−0,07	0,03	41,99	−2,25	0,15	ns
IgG	LIP5a	Uninfected vs High	−0,07	0,04	40,36	−2,03	0,15	ns
IgG	LIP04	Uninfected vs Medium	−0,06	0,04	41,21	−1,67	0,15	ns
IgG	SPI01	Uninfected vs High	−0,08	0,04	39,56	−1,78	0,21	ns
IgG	LIP04×3	Uninfected vs Medium	−0,06	0,04	41,97	−1,58	0,22	ns
IgG	CRT39	Uninfected vs Low	−0,01	0,05	39,87	−0,29	0,77	ns
IgG	SPI1a	Uninfected vs Low	−0,01	0,05	40,59	−0,21	0,84	ns
IgG	LIP04	Uninfected vs Low	−0,01	0,06	41,65	−0,12	0,90	ns
IgG	LIP04×3	Uninfected vs Low	−0,01	0,06	39,94	−0,09	0,93	ns
IgG	SPI01	Uninfected vs Low	0,00	0,06	39,21	−0,08	0,94	ns
IgG	SPI11	Uninfected vs Low	0,00	0,05	41,65	−0,08	0,94	ns
IgG	CRT42	Uninfected vs Low	0,00	0,05	40,02	−0,07	0,95	ns
IgG	LIP5a	Uninfected vs Low	−0,01	0,05	40,11	−0,12	0,96	ns

## Data Availability

Source data are provided with this paper.
